# Glycomics Analysis of Mammalian Heparan Sulfates Modified by the Human Extracellular Sulfatase HSulf2

**DOI:** 10.1371/journal.pone.0016689

**Published:** 2011-02-08

**Authors:** Gregory O. Staples, Xiaofeng Shi, Joseph Zaia

**Affiliations:** Department of Biochemistry, Center for Biomedical Mass Spectrometry, Boston University School of Medicine, Boston, Massachusetts, United States of America; Auburn University, United States of America

## Abstract

**Background:**

The Sulfs are a family of endosulfatases that selectively modify the *6O-*sulfation state of cell-surface heparan sulfate (HS) molecules. Sulfs serve as modulators of cell-signaling events because the changes they induce alter the cell surface co-receptor functions of HS chains. A variety of studies have been aimed at understanding how Sulfs modify HS structure, and many of these studies utilize Sulf knockout cell lines as the source for the HS used in the experiments. However, genetic manipulation of Sulfs has been shown to alter the expression levels of HS biosynthetic enzymes, and in these cases an assessment of the fine structural changes induced solely by Sulf enzymatic activity is not possible. Therefore, the present work aims to extend the understanding of substrate specificities of HSulf2 using *in vitro* experiments to compare HSulf2 activities on HS from different organ tissues.

**Methodology/Principal Findings:**

To further the understanding of Sulf enzymatic activity, we conducted *in vitro* experiments where a variety of mammalian HS substrates were modified by recombinant human Sulf2 (HSulf2). Subsequent to treatment with HSulf2, the HS samples were exhaustively depolymerized and analyzed using size-exclusion liquid chromatography-mass spectrometry (SEC-LC/MS). We found that HSulf2 activity was highly dependent on the structural features of the HS substrate. Additionally, we characterized, for the first time, the activity of HSulf2 on the non-reducing end (NRE) of HS chains. The results indicate that the action pattern of HSulf2 at the NRE is different compared to internally within the HS chain.

**Conclusions/Significance:**

The results of the present study indicate that the activity of Sulfs is dependent on the unique structural features of the HS populations that they edit. The activity of HSulf2 at HS NREs implicates the Sulfs as key regulators of this region of the chains, and concomitantly, the protein-binding events that occur there.

## Introduction

Cell surface heparan sulfate (HS) regulates a multitude of biochemical events including homeostasis, inflammation, angiogenesis, differentiation, and proliferation [1], [2]. These linear carbohydrates are expressed as a mixture of glycoforms of varying length and primary sequence [3]. The complexity of HS chains is derived from their non-template driven biosynthesis, but despite this, certain structural elements remain consistent in HS populations from a given tissue among different individuals [4]. How the expression of conserved structural elements is controlled is not clear from the enzymatic steps that assemble HS. Biosynthesis begins in the Golgi by addition of alternating GlcNAc and GlcA units to a linker tetrasaccharide on a core protein [5]. The elongating chains are modified by replacement of a subset of *N-*acetates with *N-*sulfate and epimerization of some GlcA residues to IdoA. *O-*sulfotransferases also modify the chains, most commonly at the *2O-*position of IdoA and the *6O-*positions of GlcNAc and GlcNS. The end result is a domain structure consisting of regions of high *N-*sulfation (NS domains), high *N-*acetylation (NA domains), and alternating *N-*substitutions (NA/NS domains). It is the structure and organization of these domains, particularly the NS domains [6], that is responsible for the protein-binding abilities of HS. The modifications conferred during HS biosynthesis were thought to be the sole determinants of HS structure until the discovery of the cell surface HS editing enzymes known as the Sulfs [7].

The avian enzyme QSulf1 was the first Sulf family member to be identified, and the importance of this discovery was underscored by the ability of the enzyme to modulate Wnt signaling in developing quail embryos [8]. QSulf1 possesses a sequence with high homology to the catalytic domain of GlcNR6ase, a lysosomal glucosamine exosulfatase that functions in *6O-*desulfation during HS degradation. Subsequently, murine and human Sulf1 isoforms were identified, in addition to a related family member, Sulf2 [9]. These enzymes were shown to catalyze removal of *6O-*sulfate groups from glucosamine residues of heparin with maximal activity near neutral pH. Despite the homology of the Sulfs to GlcNR6ase, there were a number of key differences that carried important biochemical implications. First, the Sulfs were shown to be endo-, rather than exosulfatases [9]. Additionally, they had an *N*-terminal secretion signal peptide and contained a unique hydrophilic domain that could interact with cell surface components [8]. The hydrophilic domain was shown to be a critical component for the function of the Sulfs, in that it was essential for cell-surface attachment, substrate binding, and catalytic activity [10].

A number of studies have characterized the context in which Sulfs edit the *6O-*sulfation pattern of HS. QSulf1 was shown to release *6O-*sulfate from both IdoA2S-GlcNS6S and GlcA-GlcNS6S, disaccharides known to originate from NS domains [11]. IdoA-GlcNS6S, however, was not a QSulf1 target, nor were any *6O-*sulfates located in NA/NS domains [12]. Investigations into mammalian Sulfs have yielded slightly different substrate specificities. For example, the murine Sulfs have been shown to alter the *6O-*sulfation state of NA/NS domains of HS *in vivo* [13], [14], but not *in vitro* [14].

The release of *6O-*sulfates by the Sulfs with respect to the total sulfate content of HS (the sum of all *N-* and *O-*sulfates), has been estimated as 5–7% from *in vitro* experiments using QSulf1 [15]. These changes, though relatively small in magnitude, have been associated with alterations in the binding of HS to extracellular signaling molecules. In the cases of GDNF [16], BMP [12], Shh [17], [18], and Wnt [15], [19], Sulfs serve as positive regulators and enhance signaling. The opposite is true for FGF2 [14], [20], HB-EGF [21], HGF [22], and TGF-β [23]. The long list of important growth factors and morphogens, the activities of which are modulated by the Sulfs, indicates the important roles of these extracellular HS editors in proliferation, migration, and differentiation. Depending on the context, Sulfs can serve as oncogenic effectors or tumor suppressors, and reports detailing the role of Sulfs in cancer are numerous [21], [22], [24], [25], [26], [27]. Most recently, Sulf2 was identified as a transcriptional target of the tumor suppressor p53 [28].

In light of findings that changes in Sulf expression levels influence HS biosynthesis [29], the aim of the current study was to extend knowledge of Sulf modification of HS by analyzing various HS substrates that have been modified by recombinant human Sulf2 (HSulf2). These studies aimed to understand how Sulfs edit HS isolated from different a certain tissue contexts, and the use of a purified enzyme preparation eliminated the variables, such as altered expression of HS biosynthetic enzymes, that confounded the study of Sulf activity in Sulf over-expressing or knockout animals or cell lines. The current experiments also provided the first assessment of HSulf2 activity at HS chain termini, regions that have been implicated in biochemically important protein-binding events that control developmental processes.

## Results

The activity of HSulf2 on various bovine HS substrates was examined by disaccharide analysis using size-exclusion liquid chromatography-mass spectrometry (SEC-LC/MS), a technique that has previously been applied to the analysis of HS disaccharides from mammalian organs [30], [31]. The bovine samples included HS from lung, intestine, aorta, and two fractions from kidney that were purified using two different concentrations of salt (1.1 M or 1.25 M NaCl) for elution from anion exchange chromatography [11]. The samples were digested exhaustively with HSulf2 and then subjected to exhaustive depolymerization using a mixture of heparin lyases I, II, and III. These conditions generate an array of disaccharides, designated subsequently using the structural code of Lawrence and Esko [32]. This alphanumeric code (see Fig. S1) consists of four digits that designate the structures of the uronic acid and glucosamine that comprise each disaccharide. The first position specifies whether the uronic acid is saturated (U) or Δ^4,5^-unsaturated (D). The second digit specifies whether the uronic acid is unmodified (0) or modified by *2O-*sulfation (2). The third digit specifies whether the glucosamine is *N-*acetylated (A) or *N-*sulfated (S). The fourth digit specifies whether the glucosamine is unmodified (0) or modified by *6O-*sulfation (6). For example, D2S0 specifies the structure delta-uronic acid-2-sulfate-*N-*sulfoglucosamine and U0A6 specifies the structure uronic acid-*N*-acetylglucosamine-6-sulfate.

The SEC LC/MS method generated disaccharide analysis profiles consistent with those from other methods while producing additional information on NRE disaccharides and low abundance disaccharides containing an unmodified glucosamine primary amino group. For example, the SEC LC/MS method measured the abundance of the D2S6 disaccharide in rat brain as 4.2±0.1% [33]. This value is consistent with the 3.9-5.4% range obtained by others for rat brain HS [34]. Another group has found rat cerebrum and cerebellum HS to have between 6–8% of D2S6 [35]. Similar abundances are observed in mouse brain [4]. It is likely that the small differences in results obtained using SEC LC/MS and other methods arise from the samples and the biases of the tissue extraction methods used in each laboratory.

**Figure 1:**
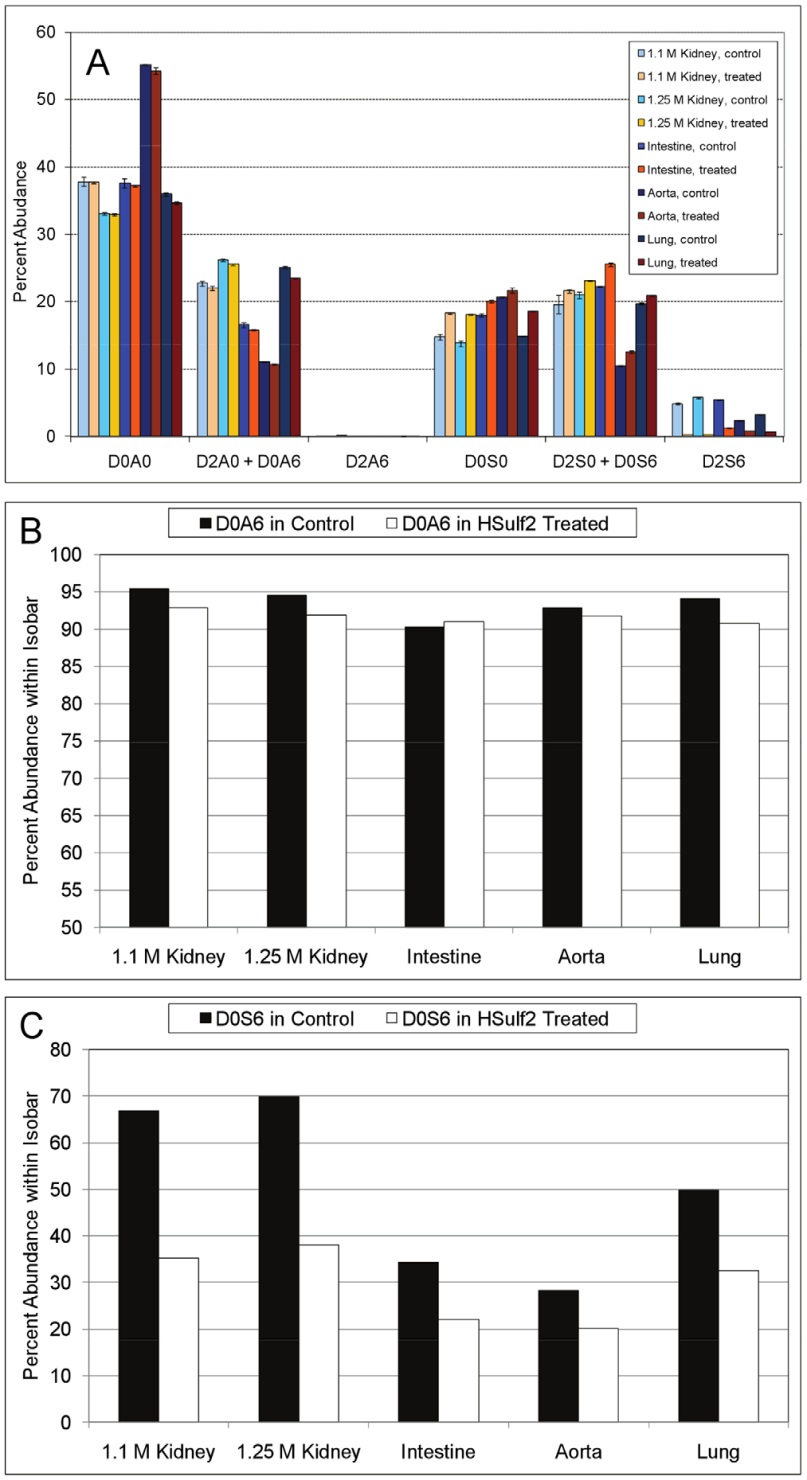
Disaccharide abundances before and after HSulf2 digestion. (A) Abundances of Δ^4,5^-unsaturated disaccharide abundances from control and HSulf2 treated HS. Peak areas were determined by integration of extracted ion chromatograms for each of the structures indicated on the X axis. Error bars are calculated from triplicate experiments and are reported as +/− S.D. Two *6O-*sulfated disaccharides, D0S6 and D0A6, occur as isomer pairs with D2S0 and D2A0, respectively. Tandem MS was used to determine the percent abundance of each *6O-*sulfated disaccharide (within its isobar), before and after HSulf2 treatment. (B) The percent abundance of D0S6 in the D2S0/D0S6 isomer pair before and after HSulf2 treatment. (C) The relative abundance of D0A6 in the D2A0/D0A6 isomer pair before and after HSulf2 treatment.

The abundances of HS Δ^4,5^-unsaturated disaccharides before and after digestion with HSulf2 as determined using SEC LC/MS are shown in Fig. 1A. The relative abundances of control or HSulf2 treated disaccharides were calculated from peak area integration of EICs of disaccharides eluting from the SEC column. As expected, HSulf2 treated HS displayed a decrease in the abundance of D2S6, the principal disaccharide substrate of the Sulfs [9], [10].

The relative changes in the abundance of two pairs of isobaric disaccharides was determined using tandem MS, following the rationale of previously developed methods [36], [37]. The data show a small decrease in the D2A0/D0A6 pair due to HSulf2 digestion (Fig. 1B). The D0A6 abundance of this pair, as determined using tandem MS, decreased in all samples except for intestine. These results demonstrate that D0A6 is a target for HSulf2. The percentage of D0S6 in the D2S0/D0S6 isomer pair decreased dramatically resulting from HSulf2 treatment, as determined using tandem mass spectrometry (Fig. 1C). The data are consistent with the conclusion that HSulf2 converts D2S6 to D2S0 and D0S6 to D0S0. The overall increase in abundance of the D2S0/D0S6 pair is due to the increase in the abundance D2S0. The abundances of D0A0 are the same before and after HSulf2 treatment for all organs. This result demonstrates that HSulf2 digestion does not bias the activity of the heparin lyase enzymes used to depolymerize HS.

**Figure 2:**
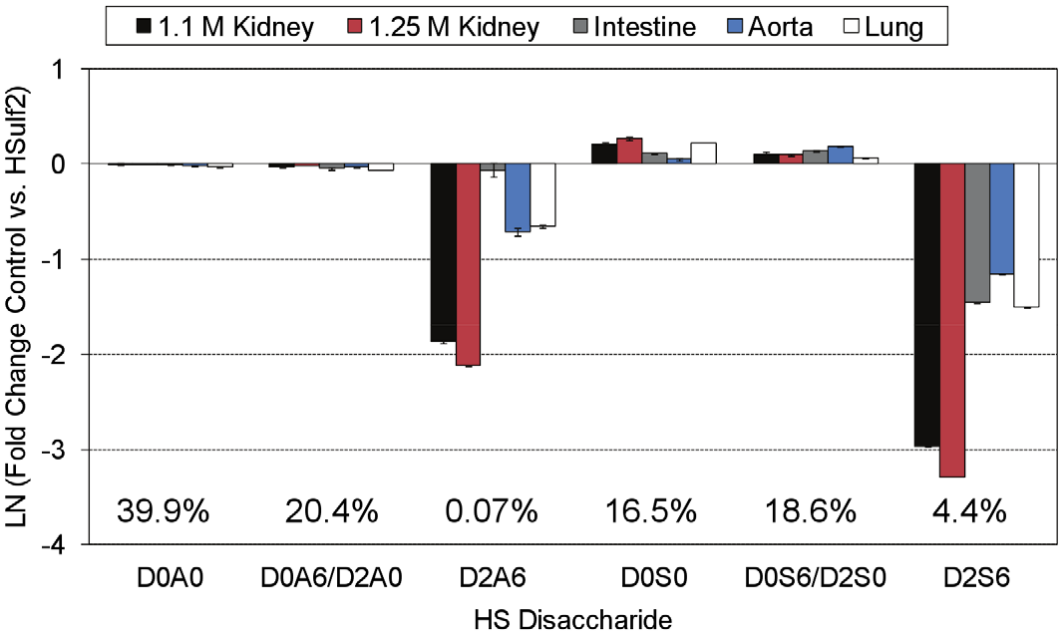
Fold change plot of the abundances of Δ^4,5^-unsaturated disaccharides from control and HSulf2 treated bovine HS samples. Disaccharide abundances from Fig. 1A are shown as fold-change differences relative to control samples. Error bars are calculated from triplicate experiments and are reported as +/− S.D.

Changes in the profile of internal, Δ^4,5^-unsaturated disaccharides from the HSulf2 treated bovine organ HS samples are shown in Fig. 2. The differences are displayed as a natural log fold-change between control and HSulf2 treated samples. The abundance of each disaccharide, averaged over the five HS sources, is shown as a percentage of all Δ^4,5^-unsaturated disaccharides on the graph. As mentioned, the trisulfated disaccharide, D2S6, decreased in abundance for all organ HS populations. This change, however, occurred in an organ specific manner, with the greatest decrease occurring for bovine 1.25 M kidney HS, and the smallest decrease occurring for bovine aorta HS. The abundance of D2A6, a disaccharide found in NA/NS domains, was also significantly decreased in the organ HS populations, with the exception of HS from bovine intestine. Two other *6O-*sulfate containing disaccharides, D0A6 and D0S6, occur as isomer pairs (with D2A0 and D2S0, respectively) in the MS mode.

**Figure 3:**
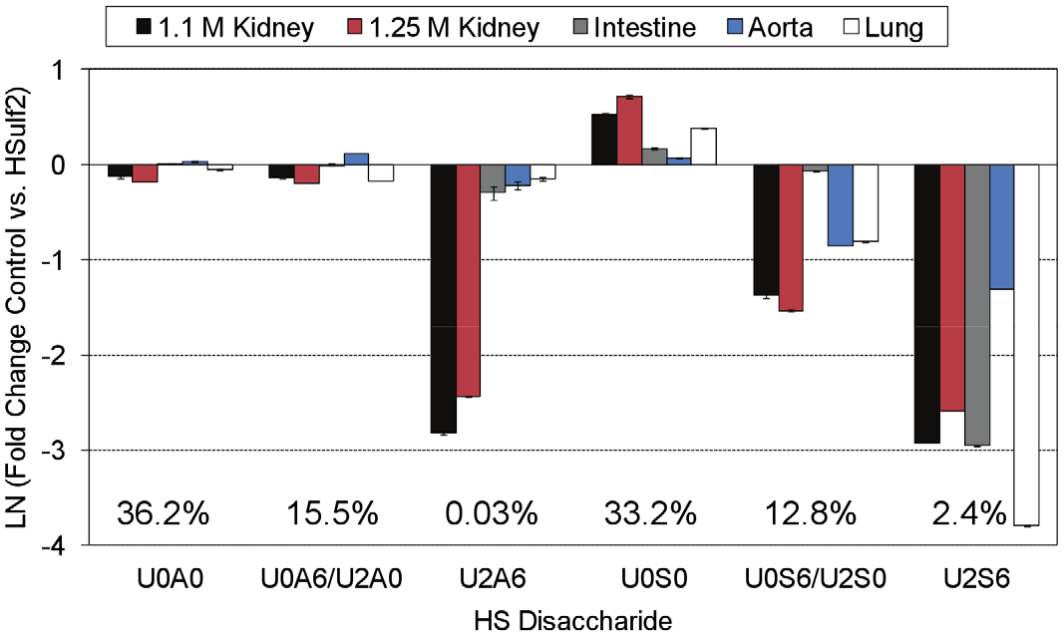
Fold change plot of the abundances of saturated disaccharides from control and HSulf2 treated bovine HS samples. Control or HSulf2 treated bovine HS samples were analyzed using SEC-MS. Changes in disaccharide abundance after HSulf2 treatment are shown as a fold-change difference. The changes in the abundance of U0A6 and U0S6 with respect to their isobars cannot be determined due to the low abundance of saturated disaccharides. Error bars are calculated from triplicate experiments and are reported as +/− S.D.

Saturated disaccharides resultant from the NRE of the bovine HS chains were also quantified using SEC-LC/MS, and differences in the abundance before and after treatment with HSulf2 are shown in Fig. 3. The abundance for each disaccharide, averaged over the five HS sources, is shown as a percent of all saturated disaccharides on the graph. This information can be used to evaluate the changes induced by HSulf2 at HS termini. The change in the amount of U2S6 (the saturated counterpart of D2S6) observed at the NRE after HSulf2 treatment differed from the change in D2S6 observed from the internal regions of the HS chains (Fig. 2). More specifically, the changes observed for U2S6 for bovine intestine and lung were much greater than for D2S6. Overall, saturated disaccharides represent a small fraction of the total disaccharides liberated by heparin lyases. The low MS ion abundances for saturated disaccharides preclude tandem MS experiments used for differentiation of disaccharides that occur as isomer pairs. Despite this, there are readily apparent differences in the profile of the U0S6/U2S0 isobar at the NRE compared to internal regions after HSulf2 treatment. The abundance of U0S6/U2S0 is lower for all samples following HSulf2 digestion, and this is accompanied by an increase in the abundance of U0S0. Again, these changes are organ specific. U2A6 was lower in abundance at the NRE for both of the bovine kidney samples, and only slightly less abundant for intestine, aorta and lung HS. An experiment using inactivated HSulf2 showed no changes in the abundances of HS disaccharides following lyase digestion, relative to samples in which no HSulf2 was added. These results show that the experimental digestion and workup conditions did not cleave or otherwise modify the HS chains.

**Figure 4:**
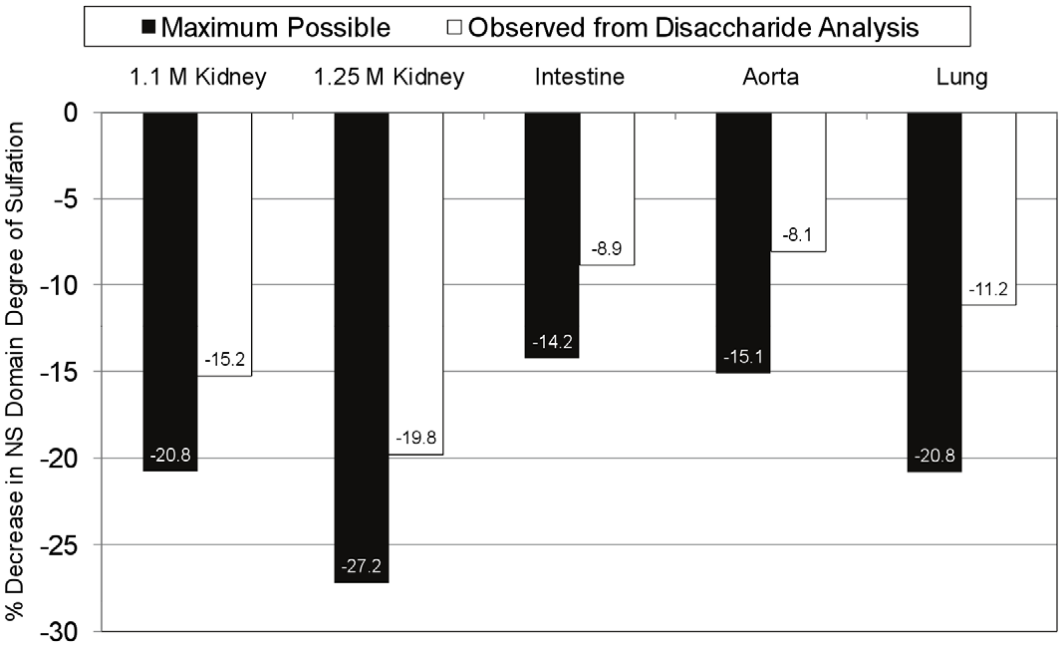
Changes in degree of sulfation of bovine HS NS domains due to HSulf2 digestion. The maximum possible change in the degree of sulfation (sulfates per disaccharide unit) of NS domains from bovine HS chains after HSulf2 treatment (black bars) was calculated, based on previously quantified nitrous acid depolymerization products generated from the same sample pool [11]. This is compared to the change in degree of sulfation calculated based on the disaccharide data generated during the current study (Fig. 1A).

Maccarana *et. al.* have previously analyzed the same pool of bovine HS samples [11] by profiling disaccharides produced from nitrous acid depolymerization. Under the conditions they used, disaccharides originating from NS domains were liberated from the HS chains. These data can be used to determine the profile of disaccharides that reside within these highly sulfated domains. We combined this information with the current data to determine the change in degree of sulfation for the NS domains of each of the bovine HS substrates that would occur upon treatment with HSulf2, as shown in Fig. 4. The black bars show the change in NS domain degree of sulfation that would occur if all NS domain *6O-*sulfates were liberated by HSulf2. The changes in degrees of sulfation of NS domains that occur based on the current disaccharide analysis (Fig. 1) are shown in the white bars in Fig. 4. In all cases, the observed change in degree of sulfation does not reach the maximum possible value. The patterns for decrease in NS domain degree of sulfation are organ specific. Additionally, there are differences between the patterns observed for the extent of change in degree of sulfation when comparing the maximum vs. observed scenarios. For example, the order of percent decrease in degree of sulfation for the observed scenario is aorta < intestine < lung <1.1 M kidney <1.25 M kidney while the order for the maximum scenario is intestine < aorta < lung/1.1 M kidney <1.25 M kidney.

**Figure 5:**
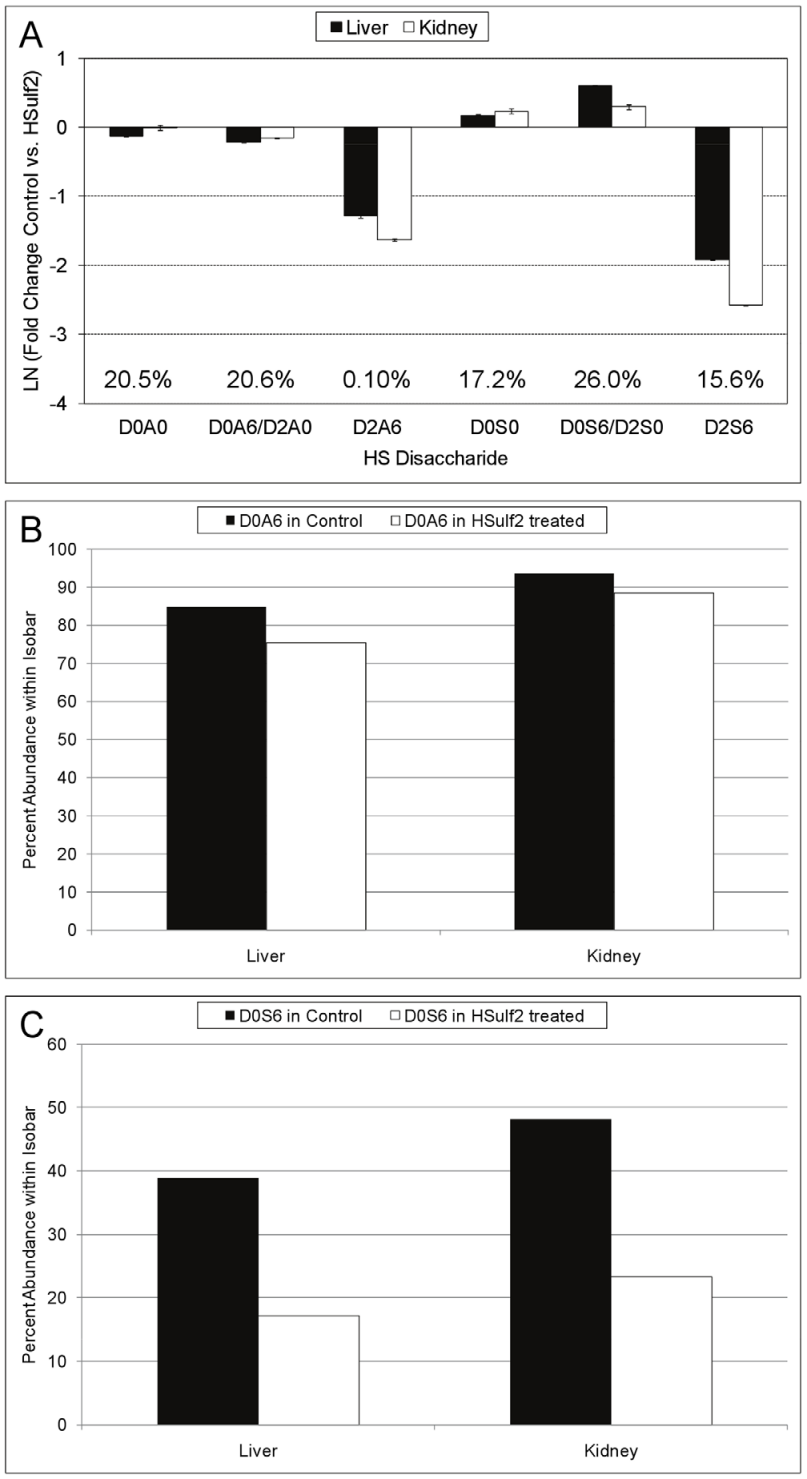
Fold change plot of the abundances of Δ^4,5^-unsaturated disaccharides from control and HSulf2 treated murine HS samples. **Control or HSulf2 treated murine HS samples were analyzed using SEC-MS.** (A) Disaccharide abundances after HSulf2 treatment are shown as a fold-change difference relative to control samples. Two other *6O-*sulfated disaccharides, D0A6 and D0S6, occur as isomer pairs with D2A0 and D2S0, respectively. Tandem MS was used to determine the percent abundance of each *6O-*sulfated disaccharide (within its isobar), before and after HSulf2 treatment. (B) The relative abundance of D0A6 before and after HSulf2 treatment. (C) The relative abundance of D0S6 before and after HSulf2 treatment. Error bars are calculated from triplicate experiments and are reported as +/− S.D.

Of additional interest was the editing of the fine structure of other mammalian HS substrates by HSulf2. These experiments would determine whether the trends we observed for the editing of bovine HS by HSulf2 were also true for HS isolated by different approaches and from different animals. To this end, murine kidney and liver HS, known to be among the most sulfated of HS populations from this organism [4], were analyzed. Murine liver has been shown to contain a very high proportion of D2S6 [4], [38], measured previously as high as 17.0% and currently as 19.4% of total disaccharide content. Changes in the profile of Δ^4,5^-unsaturated disaccharides produced from exhaustive heparin lyase digestion of murine HS with and without HSulf2 treatment are shown in Fig. 5A. The abundance of each disaccharide, averaged over the two HS sources, is shown as a percentage of all Δ^4,5^-unsaturated disaccharides on the graph. Interestingly, the pattern of change in the disaccharides after HSulf2 treatment is very similar to that observed for bovine HS (Fig. 2). Tandem MS revealed only modest desulfation of D0A6 from the murine samples (Fig. 5B), which was observed as 11.1% for liver and 5.6% for kidney. As observed for the bovine HS samples, significant desulfation of D0S6 occurred (Fig. 5C). The decreases in abundance of this disaccharide were 55.8% and 51.5% for liver and kidney, respectively.

**Figure 6:**
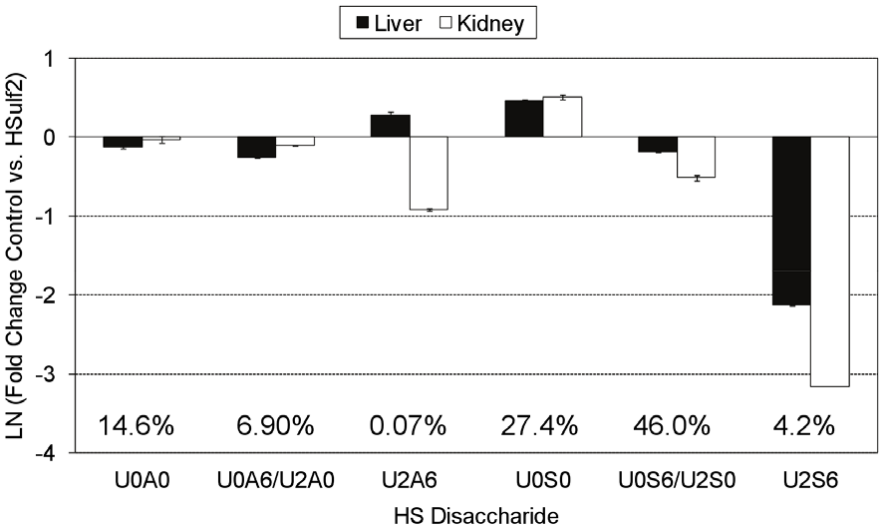
Fold change plot of the abundances of saturated disaccharides from control and HSulf2 treated murine HS samples. Control or HSulf2 treated murine HS samples were analyzed using SEC-MS. Changes in disaccharide abundance after HSulf2 treatment are shown as a fold-change difference. The changes in the abundance of U0A6 and U0S6 with respect to their isobars cannot be determined due to the low abundance of saturated disaccharides. Error bars are calculated from triplicate experiments and are reported as +/− S.D.

HSulf2 induced similar changes to the NRE of murine HS as it did to the NRE of bovine HS, as shown in Fig. 6. The abundance of each disaccharide, averaged over the two HS sources, is shown as a percentage of all saturated disaccharides on the graph. For murine kidney and liver HS, the fold change of U2S6 detected at the NRE after HSulf2 treatment is similar to that of the corresponding internal disaccharide, D2S6 (Fig. 5A). The decrease in the abundance of the U0S6/U2S0 isobar observed at the NRE of bovine organ HS chains is also present for the murine HS preparations. U2A6 decreased in abundance following HSulf2 treatment in the case of murine kidney, but not for murine liver.

**Figure 7:**
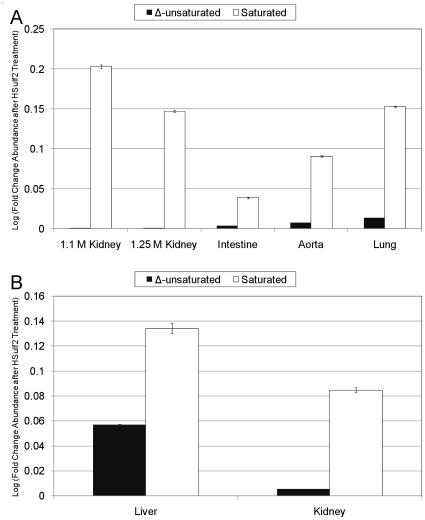
Influence of HSulf2 digestion on Δ^4,5^-unsaturated and saturated disaccharide abundances following lyase depolymerization. The abundances of total saturated and Δ^4,5^-unsaturated disaccharides in control or HSulf2 treated samples were compared and expressed as a LN fold change. (A) The fold-change in abundance of Δ^4,5^-unsaturated (black bars) or saturated (white bars) disaccharides subsequent to HSulf2 treatment of bovine HS samples. (B) The fold-change in abundance of Δ^4,5^-unsaturated (black bars) or saturated (white bars) disaccharides subsequent to HSulf2 treatment of murine HS samples. Error bars are calculated from triplicate experiments and are reported as +/− S.D.

To further characterize the changes induced at the NRE of HS by HSulf2, the total abundance of Δ^4,5^-unsaturated or saturated disaccharides was compared before and after treatment of the samples with HSulf2. The mass spectral abundances from the SEC separations are expressed as a fold change for both bovine (Fig. 7A) and murine (Fig. 7B) HS samples. Small increases in the abundance of Δ^4,5^-unsaturated disaccharides were detected following HSulf2 treatment. Comparatively large increases were observed, however, for saturated disaccharides.

**Figure 8:**
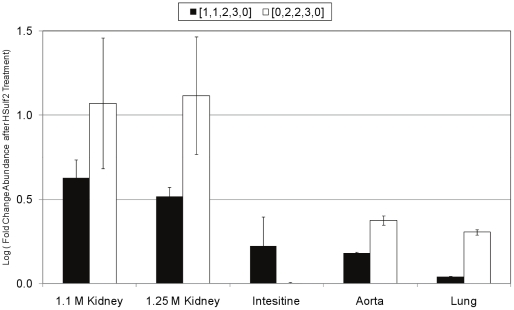
Effect of HSulf2 digestion on abundances of lyase-resistant tetrasaccharides in exhaustive digests of bovine HS. Two tetrasaccharides that resist exhaustive lyase digestion were quantified in control and HSulf2 treated bovine HS samples. The black bars show the fold change in abundance of [1,1,2,3,0]. The white bars show the fold change in abundance of [0,2,2,3,0]. Error bars are calculated from triplicate experiments and are reported as +/− S.D.

To assess the possible origin of the increase in saturated structures, lyase resistant tetrasaccharides were examined. The SEC-UV traces showed small increases in absorbance in the range of elution time that corresponds to elution of tetrasaccharides (Fig. S2). The SEC LC/MS data detected one major tetrasaccharide composition in all samples, [1,1,2,3,0] (the tetrasaccharide composition is given as [ΔHexA, Hex, GlcN, Sulfate, Acetate]), along with its saturated counterpart, [0,2,2,3,0]. The abundances of these structures were considerably higher in the bovine samples, and were too low in the murine samples for accurate quantitation. This is likely explained by the amount of HS injected onto the LC/MS system (higher for bovine samples than for murine samples, according to total ion counts) and the intensity of the tetrasaccharides was proportional to the overall signal for all disaccharides observed in the LC/MS datasets. With the exception of bovine intestine, an increase in the abundance of [1,1,2,3,0] and [0,2,2,3,0] was observed for all samples after treatment with HSulf2, as shown in Fig. 8. The abundances of [1,1,2,3,0] and [0,2,2,3,0] are observed to increase in the bovine HS populations treated with HSulf2, except for bovine intestine HS. Interestingly, when compared to its internal counterpart, [0,2,2,3,0] displayed a greater fold-change increase in abundance.

These lyase-resistant oligosaccharides represent a small percentage of domains in HS chains. It is known that disaccharide repeats containing a 3*O*-sulfated GlcN residue resist heparin lyase cleavage [39]. Such residues are required for anticoagulant function of heparin/HS. The changes in the abundances observed in Fig. 8 that result from HSulf2 digestion indicated that the presence of 6*O*-sulfate groups influences the susceptibility of the oligosaccharides to lyase digestion. The SEC LC/MS system is able to detect tetrasaccharides, while oligosaccharides of dp6 or longer cannot be detected because of their highly polydisperse nature. The data in Fig. 7 and Fig. 8 suggest that HSulf2 digestion changes the susceptibility of HS chains to lyase digestion. The increases in abundances for dp2 and dp4 in the HSulf2 digested samples indicate that the overall susceptibility of the HS chains to lyase digestion is increased by the removal of *6O-*sulfate groups. The data also show that the extent to which saturated dp2 and dp4 increase in abundance is greater than for their internal counterparts. This further suggests that HSulf2 displays increased activity towards the NRE than the internal oligosaccharides, taken as an average. The data do not rule out that there may be particular internal domains toward which HSulf2 is highly active. Thus, it appears that NRE domains have increased susceptibility towards HSulf2 relative to the average internal domains and that this pattern is present in most organs samples studied.

**Figure 9:**
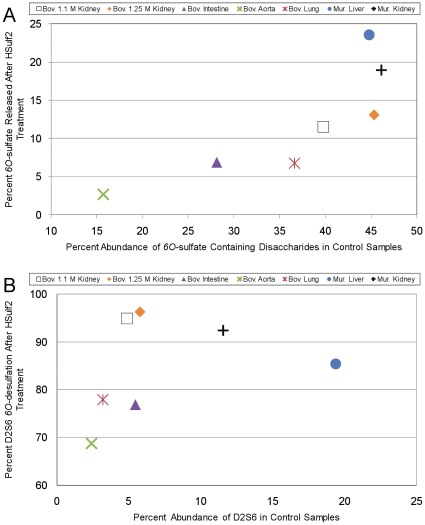
Comparison of the extent of HSulf2 mediated release of *6O-*sulfate from bovine and murine HS samples. The release of *6O-*sulfate from the various HS samples was determined by summing the decreases in abundance of *6O-*sulfated disaccharides (Fig. 1A and Fig. 5A). (A) The release of *6O-*sulfate by HSulf2 as a function of total *6O-*sulfate content in the control samples. (B) The release of *6O-*sulfate from D2S6 after HSulf2 treatment as a function of total D2S6 content in the control samples.

Fig. 9A shows the total percentage of *6O-*sulfate released from each of the bovine or murine HS preparations as a function of the total abundance of *6O-*sulfates in each chain. In general, as the *6O-*sulfate content increases, so does the amount of *6O-*sulfate released. However, while murine liver, murine kidney, and bovine 1.25 M kidney have similar total *6O-*sulfate content, there is considerable variation in the amount of *6O-*sulfate released. The ability of HSulf2 to edit HS sulfation may be assessed in more detail by examining the *6O-*desulfation of D2S6, as shown in Fig. 9B. This figure shows the percent of *6O-*desulfation of D2S6 as a function of the overall D2S6 content for a given preparation. The two bovine kidney preparations exhibit the highest degree of *6O-*desulfation despite the fact that their initial D2S6 content is much lower than that of either murine kidney or murine liver HS. This indicates that D2S6 is desulfated in a manner dependant on the domain context in which it resides.

## Discussion

Sulf1 and Sulf2 are thought to work cooperatively to regulate developmental processes by modifying HS *6O-*sulfate patterns [16], [18], [40]. Redundancy in the roles of Sulf1 and Sulf2 is evident from studies of double knock-out mice, where pups display severe defects in esophageal development that result in death [16]. In contrast, mice that are deficient in Sulf1 or Sulf2 alone survive, albeit with some developmental defects [16], . The story is complicated by the fact that in some cases, Sulf1 and Sulf2 are differentially expressed, for example in the developing mouse brain [41] and regenerating skeletal muscle [42] along with the fact that the Sulfs may have different substrate specificities *in vivo* [13]. Nonetheless, it is clear from these studies that the editing of HS fine structure by the Sulfs is essential for developmental processes.

Recently, co-regulatory functions of the Sulfs were identified in primary MEF cells. For example, up-regulation of *mSulf1* was observed in *mSulf2^−/−^* cells, as measured by real-time PCR and Northern blot analysis. Additionally, down-regulation of *mSulf2* was observed in *mSulf1^−/−^* cell lines [13]. Interestingly, these effects were not observed in immortalized MEF cells [14]. However, the immortalized cells displayed altered gene expression of HS *6O-*, *2O-*, and *3O-*sulfotransferases [14]. Loss of Sulf2 resulted in a nearly three-fold decrease in HS2ST1 expression. Currently, it is not understood how the Sulfs mediate effects on the expression of HS biosynthetic enzymes. These phenomena make it difficult to assess the structural changes in HS molecules that are induced solely by the enzymatic activity of Sulf enzymes.

The characterization of the *in vitro* alteration of *6O-*sulfation state by HSulf2 on a set of HS substrates was thus initiated. The results serve to increase the understanding of Sulf activity *in vivo*, where the biological functions of HS are determined by the organization of sulfation and epimerization modifications along the chain. Disaccharide analysis from bovine (Fig. 2) and murine (Fig. 5A) HS substrates reveals, as expected, that HSulf2 is most active on the trisulfated disaccharide species, D2S6. Another NS domain disaccharide, D0S6, is also significantly *6O-*desulfated by HSulf2. While the majority of HSulf2 activity is localized to NS domains, a measurable amount of activity was detected in NA/NS domains. *6O-*desulfation of D0A6 is observed to occur for all substrates, except for bovine intestine. Additionally, *6O-*desulfation of D2A6, a disaccharide that is typically in very low abundance in HS populations, is observed to occur for all substrates, again with the exception of bovine intestine HS. In all cases, the degree to which HSulf2 releases *6O-*sulfate from either NA/NS domains or NS domains varies from one substrate to another. This suggests that desulfation of a given *6O-*sulfate by HSulf2 is dependent on the domain structure surrounding the target sulfate moiety. This appears to be particularly true when comparing the desulfation of NS domains vs. NA/NS domains, which each contain about half of the total *6O-*sulfate content of an HS chain [11].

The current data are consistent with a recent report that the hydrophilic domain of the Sulfs binds HS chains with high affinity and in a manner dependent on *6O-*sulfation [43]. It was proposed that the specificity of this interaction is a mechanism for targeting of the catalytic domain of the Sulfs to the *6O-*sulfates of an HS chain. Additionally, it was proposed that multiple sites of contact would be needed to stabilize the interaction of the hydrophilic domain with HS chains, meaning that fine structure surrounding a given *6O-*sulfate would be a major determinant of Sulf substrate processing. These features may explain the variation we observe in the HSulf2 processing of the mammalian HS chains investigated in this study. It appears likely that the catalytic activity of Sulfs is partially instructed by the HS biosynthetic reactions that occur within a population of cells, particularly by *6O-*sulfotransferases.

A benefit of the use of MS for the current experiments is that, for the first time, an assessment of Sulf activity at the NRE of HS chains is possible. The disaccharide analysis results indicate that the NRE is acted upon by HSulf2 to a higher degree, as compared to internal regions of the chain (Fig. 3 for bovine HS and Fig. 6 for murine HS). This difference in HSulf2 action at the NREs is likely to influence protein-binding events that occur at this location. The interaction of FGF2 with FGFR and HS chains is a particularly relevant example in this case, because alterations in *6O-*sulfation state caused by disruption of *6O-*sulfotransferase expression have been shown to have drastic effects on FGF2 signaling [44]. Similarly, alteration of the *6O-*sulfation state of HS by Sulfs has been shown to affect formation of the FGF:FGFR:HS ternary complex [25]. In this case, Sulf activity serves to attenuate FGF2 signaling. One of the current models of FGF signaling implicates the NRE as a key location of FGF:HS:FGFR ternary complex formation [45], [46], [47]. In this, “cooperative end structures” model, two 1∶1∶1 FGF:HS:FGFR ternary complexes form a dimer, dependent on the NREs of separate HS chains. The altered activity of HSulf2 at the NRE of the HS substrates used in this study implicates this enzyme as a key regulator of FGF ternary complex formation at the NRE.

It will be of interest to extend the current studies to an examination of oligosaccharides derived from tissue-specific HS samples. Such oligosaccharides correspond to HS domains and their analysis will determine how Sulf activity modifies HS structure in a more global context. Additionally, MS protein-binding characterizations (akin to those already carried out for antithrombin III [48], [49]) between the hydrophilic domain of Sulfs and HS substrates will be very useful for determining the requirements for HS binding and editing by the Sulfs. This will provide further understanding of how the Sulfs modify the activity or concentration of signaling molecules at the cell surface.

## Materials and Methods

### Materials

HS from bovine aorta, lung, intestine, and two fractions from kidney that were purified using two different concentrations of salt (1.1 M or 1.25 M NaCl) for elution from anion exchange chromatography were a generous gift from Dr. Keiichi Yoshida. Murine liver and kidney HS were a generous gift from Dr. Fuming Zhang and Dr. Robert Linhardt. Recombinant HSulf2 was a generous gift from Shire Human Genetic Therapies (Cambridge, MA). Heparin lyases I, II, and III were from Ibex (Montreal, Quebec, Canada). HS disaccharide standards and all other reagents were from Sigma-Aldrich (St. Louis, MO).

### Treatment of heparan sulfate with HSulf2

HS samples, ∼50 µg each, were dissolved in 90 µL of 100 mM NaCl, 20 mM Tris, 1mM CaOAc, 1 mM MgCl_2_, pH 7.4 and a 10 µL aliquot of Sulf2, which contained 10 µg of enzyme in storage buffer (20 mM sodium phosphate, 500 mM NaCl, 10% glycerol, 0.5 mg/mL pefabloc, pH 7.0) was added. The reaction was allowed to proceed overnight at 37°C. Subsequently, the reaction was heat inactivated by boiling for 3 min and stored at −20°C prior to complete depolymerization using heparin lyases.

### Preparation of Δ^4,5^-unsaturated heparan sulfate disaccharides

Untreated and HSulf2 treated HS samples (∼10 µg each) were exhaustively digested using a mixture of 2 mIU each of heparin lyases I, II, and III. All reactions were carried out in 100 mM NaCl, 20 mM Tris, 1mM CaOAc, 1 mM MgCl_2_, pH 7.4. Another aliquot of heparin lyases was added after two hours of incubation and the reaction was allowed to proceed overnight. The reaction was terminated by heat inactivation.

### SEC-LC/MS analysis of HSulf2 treated heparan sulfate disaccharides

Heparin lyase depolymerized control or HSulf2 treated HS samples were analyzed directly after digestion using SEC-LC/MS [30]. Briefly, HS samples (∼1 µg per replicate) were injected onto a Superdex Peptide PC 3.2/30 column (GE Biosciences, Piscataway, NJ) online with an Applied Biosystems/MDS Sciex QSTAR Pulsar Qq-TOF mass spectrometer operating in the negative-ion mode. The isocratic mobile phase was 12.5 mM formic acid, titrated to pH 4.4 with ammonia, in 10% acetonitrile. The SEC LC/MS system is able to detect disaccharides quantities as low as 100 femtomole and perform routine quantitation of disaccharides from 1 pmol to 1 nmol. To differentiate isomeric disaccharides, tandem SEC-LC/MS experiments were performed. These experiments were separate from the runs used for glycoform quantitation described above, and utilized principles developed in previous studies for distinguishing isomeric HS or chondroitin sulfate structures [36], [37]. Commercial disaccharides were used as quantitative standards for the tandem mass spectra. Briefly, mock binary mixtures of D0A6/D2A0 and D0S6/D2S0 were prepared in ratios of 3∶0, 3∶1, 2∶1, 1∶1, 1∶2, 1∶3, 0∶3. Tandem mass spectra were acquired using a collision energy of 40 V. The signals were averaged across the most abundant region of the chromatographic peak (±0.75 min of the center). Diagnostic ions were selected for each pair of isomers. For HS D0A6/D2A0: m/z 139 and 157, respectively; for D0S6/D2S0: m/z 157 and 199, respectively. The intensities of each diagnostic ion were normalized to the sum of the signal for a given isomer pair. The data for each binary mixture were plotted, and exponential regression was used to fit a curve to the data using Igor Pro 4.07 (Wavemetrics, Portland, OR). Tandem SEC-LC/MS experiments were carried out for all control and HSulf2 treated samples. The relative abundances of the diagnostic ions of each isomeric pair were obtained and were substituted into the fitted equation to produce the relative contribution of a disaccharide to its isobar.

## Supporting Information

Figure S1 Codes for disaccharides used in this work. The monosaccharides residues are defined using the symbols shown at the bottom of the figure. The position(s) of sulfation on each monosaccharide residue are show by the text above or below the symbol. 2S, sulfation at the 2-position of HexA or ΔHexA; NS, *N*-sulfation of GlcN; 6S, sulfation at the 6-position of GlcNAc or GlcN.
(DOC)

Figure S2 SEC-UV 232 nm chromatographic traces for heparin lyase depolymerized bovine kidney HS with and without prior HSulf2 digestion.
(DOC)
