# Supplementary figures and images for: Glycomics Analysis of Mammalian Heparan Sulfates Modified by the Human Extracellular Sulfatase HSulf2
Source: PLoS One. 2011 Feb 8;6(2):e16689. doi: 10.1371/journal.pone.0016689 (PMC3035651; doi:10.1371/journal.pone.0016689)

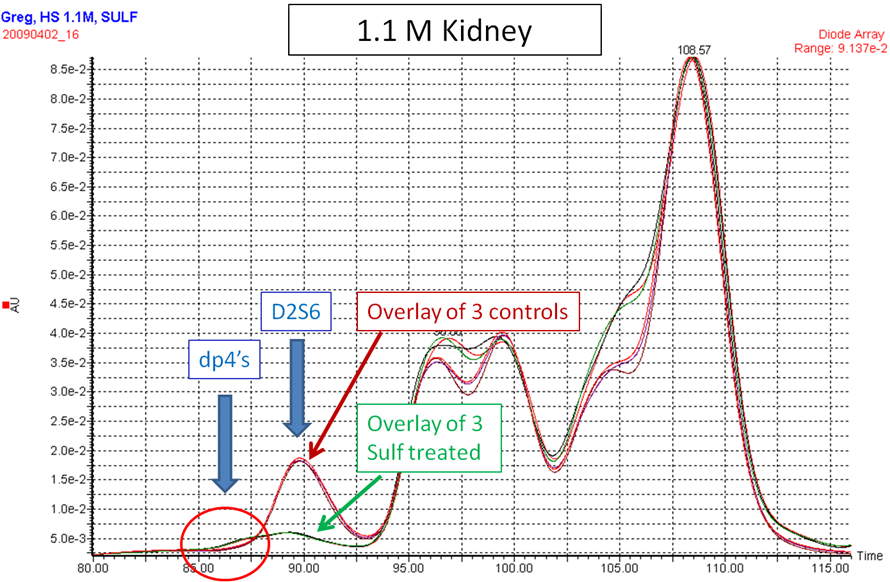

Supplement: Figure S2 — SEC-UV 232 nm chromatographic traces for heparin lyase depolymerized bovine kidney HS with and without prior HSulf2 digestion. (DOC) [file pone.0016689.s002.doc]
