# Supplementary material for: Glycomics Analysis of Mammalian Heparan Sulfates Modified by the Human Extracellular Sulfatase HSulf2
Source: PLoS One. 2011 Feb 8;6(2):e16689. doi: 10.1371/journal.pone.0016689 (PMC3035651; doi:10.1371/journal.pone.0016689)
Supplement: Supplementary file 2 [file pone.0016689.s001.doc]

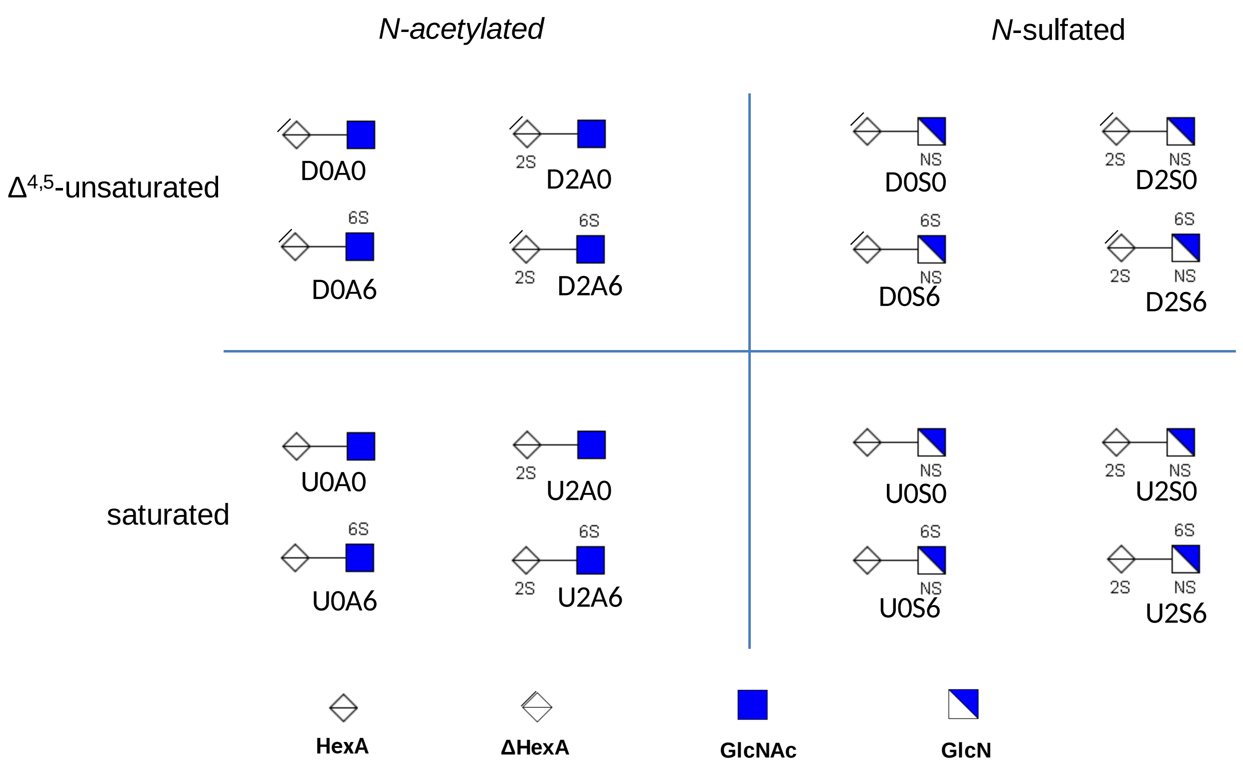
N-acetylated
N-sulfated
Δ4,5-unsaturated
saturated
D0A0
D2A0
D0A6
D2A6
D0S0
D2S0
D0S6
D2S6
U0A0
U2A0
U0A6
U2A6
U0S0
U2S0
U0S6
U2S6
2S
6S
NS
HexA
ΔHexA
GlcNAc
GlcN
